# HIV-1 Tat-induced diarrhea is improved by the PPARalpha agonist, palmitoylethanolamide, by suppressing the activation of enteric glia

**DOI:** 10.1186/s12974-018-1126-4

**Published:** 2018-03-24

**Authors:** Giovanni Sarnelli, Luisa Seguella, Marcella Pesce, Jie Lu, Stefano Gigli, Eugenia Bruzzese, Roberta Lattanzi, Alessandra D’Alessandro, Rosario Cuomo, Luca Steardo, Giuseppe Esposito

**Affiliations:** 1 Department of Clinical Medicine and Surgery, “Federico II” University of Naples, 80131 Naples, Italy; 2 Department of Physiology and Pharmacology, “Vittorio Erspamer”, La Sapienza University of Rome, 00185 Rome, Italy; 3 Department of Anatomy, China Medical University, Shenyang, Liaoning Province 110122 People’s Republic of China; 4 Department of Translational Medical Science, Section of Pediatrics, University Federico II, 80131 Naples, Italy

**Keywords:** HIV-1 Tat protein, EGCs, Diarrhea, Neuroinflammation, PEA

## Abstract

**Background:**

Diarrhea is a severe complication in HIV-1-infected patients with Trans-activator of transcription (HIV-1 Tat) protein being recognized as a major underlying cause. Beside its direct enterotoxic effects, Tat protein has been recently shown to affect enteric glial cell (EGC) activity. EGCs regulate intestinal inflammatory responses by secreting pro-inflammatory molecules; nonetheless, they might also release immune-regulatory factors, as palmytoilethanolamide (PEA), which exerts anti-inflammatory effects by activating PPARα receptors. We aimed at clarifying whether EGCs are involved in HIV-1 Tat-induced diarrhea and if PEA exerts antidiarrheal activity.

**Methods:**

Diarrhea was induced by intracolonic administration of HIV-1 Tat protein in rats at day 1. PEA alone or in the presence of peroxisome proliferator-activated receptor (PPAR) antagonists was given intraperitoneally from day 2 to day 7. S100B, iNOS, NF-kappaB, TLR4 and GFAP expression were evaluated in submucosal plexi, while S100B and NO levels were measured in EGC submucosal plexi lysates, respectively. To verify whether PEA effects were PPARα-mediated, PPARα^−/−^ mice were also used. After 7 days from diarrhea induction, endogenous PEA levels were measured in submucosal plexi homogenates deriving from rats and PPARα^−/−^ mice.

**Results:**

HIV-1 Tat protein induced rapid onset diarrhea alongside with a significant activation of EGCs. Tat administration significantly increased all hallmarks of neuroinflammation by triggering TLR4 and NF-kappaB activation and S100B and iNOS expression. Endogenous PEA levels were increased following HIV-1 Tat exposure in both wildtype and knockout animals. In PPARα^−/−^ mice, PEA displayed no effects. In wildtype rats, PEA, via PPARα-dependent mechanism, resulted in a significant antidiarrheal activity in parallel with marked reduction of EGC-sustained neuroinflammation.

**Conclusions:**

EGCs mediate HIV-1 Tat-induced diarrhea by sustaining the intestinal neuroinflammatory response. These effects are regulated by PEA through a selective PPARα-dependent mechanism. PEA might be considered as an adjuvant therapy in HIV-1-induced diarrhea.

**Electronic supplementary material:**

The online version of this article (10.1186/s12974-018-1126-4) contains supplementary material, which is available to authorized users.

## Background

The use of combined anti-retroviral therapy against human immunodeficiency virus-type 1 (HIV-1) infection has dramatically improved the survival and prognosis of patients affected by the acquired immunodeficiency syndrome (AIDS) [1]. However, chronic diarrhea is reported in up to 30% of HIV-1-infected patients and significantly contributes to AIDS morbidity [2, 3].

Many of the pathogenic effects of HIV-1 in the gut are caused by the HIV-1 trans-activating factor protein (Tat), a viral protein of 86 aminoacids, which is essential to viral replication [4]. HIV-1 Tat targets enterocytes and induces the expression of many genes regulating cells’ survival and growth; but it also affects immune and inflammatory responses, altering the intracellular calcium concentration, inducing epithelial cell apoptosis, and ultimately causing secretory diarrhea [5, 6]. The intestinal epithelial mucosa has been considered for years as the key target in HIV-1 Tat-related enterotoxicity; however, the enteric nervous system (ENS) is now emerging to be also involved [7, 8]. Within the ENS, enteric glial cells (EGCs), together with neurons, cooperate to finely regulate secretion, motility, and blood flow, as well as immune responses [9–11]. EGCs express Toll-like receptors (TLRs), and there is mounting evidence suggesting that they actively participate to the homeostatic immune control of the gut [12]. EGCs are able to secrete pro-inflammatory mediators, interleukins, and enteroglial-released factors [13–15], but they also release a number of protective mediators that sustain epithelial barrier functions [16–18]. Among EGC-derived factors, S100B, a specific glial Ca^+ 2^/Zn^+ 2^-binding protein, and nitric oxide (NO) deriving from the inducible isoform of nitric oxide synthase (iNOS) expressed by EGCs play a central role during immune-inflammatory responses [14, 19, 20]. We recently demonstrated that glial cells participate to HIV-1 Tat-induced intestinal and neurological pathogenesis [21], but the possibility to pharmacologically modulate Tat-induced secretory diarrhea by inhibiting EGC activation has not been explored yet.

Palmitoylethanolamide (PEA), an endogenous, *on-demand* released N-Acylethanolamide [22–24], exerts immunoregulatory functions targeting EGC activation in ulcerative colitis, with a consistent protection of colonic epithelial mucosa [25]. Different studies have shown that the pharmacological activity of PEA depends on its capacity to selectively bind peroxisome proliferator-activated receptor-α (PPARα), a member of a nuclear hormone receptor superfamily of ligand-activated transcription factor [24]. The involvement of and the ability of PEA to protect against HIV-1 Tat-induced diarrhea have never been investigated.

The aims of the present study were to investigate the involvement of EGCs in a rat model of diarrhea induced by the intracolonic administration of HIV-1 Tat, to characterize the mediators secreted by EGCs activation, and to evaluate the protective effect of PEA, its site, and mechanisms of action, respectively.

## Methods

### Animals and experimental design

Eight-week-old Wistar male rats (Harlan Laboratories, Udine, Italy) and 6-week-old PPARα^−/−^ mice (Taconic, Germantown, New York, USA) were used for experiments. All procedures were approved by La Sapienza University’s Ethics Committee. Animal care was in compliance with the IASP and European Community (EC L358/1 18/12/86) guidelines on the use and protection of animals in experimental research. Rats were randomly divided into the following groups (*n* = 8 each): non-diarrhea, vehicle group; HIV-1 Tat protein-induced diarrhea group; HIV-1 Tat protein-induced diarrhea group receiving daily PEA 2 and 10 mg/Kg, respectively; HIV-1 Tat protein-induced diarrhea group receiving daily PEA (10 mg/Kg) and selective PPARα antagonist MK866 (10 mg/Kg) and selective PPARγ antagonist GW9662 (1 mg/Kg), respectively; HIV-1 Tat protein-induced diarrhea group receiving 0.03% *w*/*v* lidocaine; bisacodyl group (20 mg/Kg) as internal control in some experiments. Analogously, PPARα^−/−^ mice were randomly divided into the following groups (*n* = 8 each): non-diarrhea, vehicle group; HIV-1 Tat protein-induced diarrhea group; HIV-1 Tat protein-induced diarrhea group receiving daily PEA 50 and 100 mg/Kg, respectively; HIV-1 Tat protein-induced diarrhea group receiving 0.03% *w*/*v* lidocaine; bisacodyl group (20 mg/Kg) as internal control in some experiments.

Diarrhea was experimentally induced by intracolonic administration of HIV-1 Tat protein (130 ng/Kg) at day 1. HIV-1 Tat was dissolved in pyrogen-free distillated water and a volume of 400 μl or 40 μl of a 100 ng/ml solution of HIV-1 Tat was injected into the lumen of the rat and PPARα^−/−^ mice colon (3–4 cm proximal to anus) by using a 24-gauge catheter, respectively. PEA alone, or combined with PPARs antagonists, was given intraperitoneally from day 1 to day 7.

In a subset of experiments, lidocaine hydrochloride monohydrate (Sigma-Aldrich, Milan, Italy, 0.03% *w*/*v*) dissolved in sterile, pyrogen-free distilled water, was given in a single dose through intracolonic administration at day 1 concomitantly with HIV-1 Tat. In another set of experiments, bisacodyl was administrated orally as aqueous solution at day 1 and served as positive internal control. Depending upon the experimental plan, at day 7, animals were euthanatized and colon was isolated to perform macroscopic, histochemical, and biochemical analyses as described below.

### Evaluation of diarrhea

Depending upon the experimental protocol, animals were separated in subgroups and placed separately in cages lined with filter paper to evaluate diarrhea severity. The individual cages were inspected every 2 h for 16 h, from day 1 to day 7 after HIV 1-Tat intracolonic administration, for the presence of characteristic wet diarrhoeal droppings. Daily defecation frequency and number of unformed water fecal pellets of each animal were assessed and compared with the score from the vehicle group. The data were expressed as a daily mean score (16 h) of diarrhoeal dropping number and total number of fecal pellets/wet spots for defecation frequency within 7 days from diarrhea induction. Evaluation of accumulation of intracolonic fluid was performed using the enteropooling technique according to previously described method [26]. Briefly, enteropooling is defined as the intraluminal accumulation of fluid into the small intestine and corresponds with the fluid already located in the lumen and excreted from the blood. According to the experimental plan, the entire small intestine and colon of rats and PPARα^−/−^ mice were isolated, taken care to avoid tissue rupture and loss of fluid, by removing the mesentery and connective tissue. To normalize the data, fluid accumulation was expressed as follows:

$$ \left({W}_1-{W}_2\right)/{W}_2\times 1{0}^{-6} $$

where *W*_1_ is the weight of the intestine after excision and *W*_2_ is the weight of the intestine after expulsion of its content. Water content was measured and compared with the score from vehicle group.

### Tissue preparation

To isolate submucosal plexi from animals at day 7 after diarrhea induction, we performed a slightly modified method than previously described procedure by Cirillo et al. [14]. Following dissection, colonic segments (approximately 2-cm long) were collected and placed in a cold oxygenated sterile Krebs solution containing (in mM) 117 NaCl, 4.7 KCl, 1.2 MgCl_2_ 6 H_2_O, 1.2 NaH_2_PO_4_, 25 NaHCO_3_, 2.5 CaCl_2_ 2 H_2_O, and 11 glucose under carbogen (5% CO_2_, 95% O_2_) atmosphere equilibrated at pH 7.4. The tissue was longitudinally cut along the mesenteric border, and the submucosal plexus was carefully separated from the mucosal and the muscle layers by microdissection. After removal, submucosal plexi were processed for biochemical and immunofluorescence assays.

### Protein extraction and western blot analysis

Proteins were extracted from submucosal plexi deriving from rats and PPARα^−/−^ mice at day 7 after diarrhea induction. The tissue was homogenized in ice-cold hypotonic lysis buffer to obtain cytosolic extracts and underwent electrophoresis through a polyacrilamide minigel. Proteins were transferred into nitrocellulose membrane that were saturated with non-fat dry milk and then incubated with either mouse anti-S100B (Neo-Marker, Milan, Italy), mouse anti-iNOS, rabbit anti-GFAP, rabbit anti-TLR4, and mouse anti-β-actin (all Santa Cruz Biotechnology, Santa Cruz, California, USA). Membranes were then incubated with the specific secondary antibodies conjugated to horseradish peroxidase (Dako, Milan, Italy). Immune complexes were revealed by enhanced chemiluminescence detection reagents (Amersham Biosciences, Milan, Italy). Blots were analyzed by scanning densitometry (GS-700 imaging densitometer; Bio-Rad). Results were expressed as OD (arbitrary units; mm^2^) and normalized on the expression of the housekeeping protein β-actin.

### Electrophoretic mobility shift assay (EMSA)

EMSA was performed to detect NF-kappaB activation in submucosal plexi obtained from rats and PPARα^−/−^ mice at day 7 after diarrhea induction. Double-stranded oligonucleotides containing the NF-kappaB recognition sequence for rats (5–CAACGG CAGGGGAATCTCCCTCTCCTT-3) and mice (5-TCAGAGGGGACTTTCCGAGAGG-3) were end-labeled with ^32^Pγ-ATP. Nuclear extracts were incubated for 15 min with radiolabeled oligonucleotides (2.5–5.0 × 10^4^ cpm) in 20 ml reaction buffer containing 2 mg poly dI-dC, 10 mM Tris–HCl (pH 7.5), 100 mM NaCl, 1 mM ethylenediaminetetraacetic acid, 1 mM dl-dithiothreitol, 1 mg/ml bovine serum albumin, and 10% (*v*/*v*) glycerol. Nuclear protein-oligonucleotide complexes were resolved by electrophoresis on a 6% non-denaturing polyacrylamide gel in 1 Tris Borate ethylenediaminetetraacetic acid buffer at 150 V for 2 h at 4 °C. The gel was dried and autoradiographed with an intensifying screen at − 80 °C for 20 h. Subsequently, the relative bands were quantified by densitometric scanning with Versadoc (Bio-Rad Laboratories) and a computer program (Quantity One Software, Bio-Rad Laboratories). ^32^P-γ-ATP was from Amersham (Milan, Italy). Poly dI-dC was from Boehringer-Mannheim (Milan, Italy). Oligonucleotide synthesis was performed to our specifications by Tib Molbiol (Boehringer-Mannheim).

### NO quantification

NO was measured as nitrite (NO_2_^−^) accumulation in submucosal plexi homogenates deriving from rats and PPARα^−/−^ mice at day 7 after diarrhea induction, by a spectrophotometer assay based on the Griess reaction. Briefly, Griess reagent (1% sulphanilamide, 0.1% naphthylethylenediamine in phosphoric acid) was added to an equal volume of supernatant, and the absorbance was measured at 550 nm. Nitrite concentration (nM) was thus determined using a standard curve of sodium nitrite.

### Enzyme-linked immunosorbent assay for S100B

Enzyme-linked immunosorbent assay (ELISA) for S100B (Biovendor R&D, Brno, Czech Republic) was carried out on submucosal plexi lysates obtained from rats and PPARα^−/−^ mice at day 7 after diarrhea induction, according to the manufacturer’s protocol. Absorbance was measured on a microtiter plate reader. S100B level was determined using standard curves method.

### Immunofluorescence analysis

Additional experiments were performed using specific isotype antibody controls (Abcam, Cambridge, UK), at the same concentration as the primary antibodies. Tissues were then incubated in the dark with the proper secondary antibody: fluorescein isothiocyanate-conjugated anti-rabbit or Texas Red-conjugated anti-mouse, respectively (both Jackson ImmunoResearch Laboratories, West Grove, PA, USA). Tissues were analyzed with a microscope (Nikon Eclipse 80i), and images were captured by a high-resolution digital camera (Nikon Digital Sight DS-U1).

### Measurement of PEA in rats and mice EGCs

Tissue content of endogenous PEA was measured in submucosal plexi homogenates deriving from both rats and PPARα^−/−^ mice at day 7 after diarrhea induction. Following isolation of lipidic fraction by tissue homogenates, intracellular PEA concentrations (pmol) were normalized per milligram of extracted lipid fraction and were analyzed by liquid chromatography coupled to tandem mass spectrometry (LC-MS/MS) using a 325-MS LC/MS Triple Quadrupole Mass Spectrometer (Agilent Technologies Italia, Cernusco s/N, Italy) according to literature [27].

### Statistical analysis

Results were expressed as mean ± SEM of *n* experiments. A statistical analysis was performed using parametric one-way analysis of variance (ANOVA), and multiple comparisons were performed by Bonferroni’s post hoc test; *p* values < 0.05 were considered significant.

## Results

### Intracolonic administration of HIV-1 Tat induces diarrhea in rats and stimulates the release of endogenous PEA

**Fig. 1:**
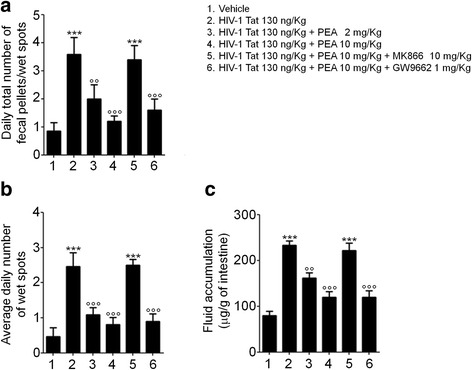
Palmitoylethanolamide (PEA) improves diarrheal hallmarks in rats via PPARα activation. Intracolonic administration of HIV-1 Tat protein (130 ng/Kg) resulted in a significant increase of **a** daily defecation frequency, **b** average daily number of wet spots, and **c** fluid accumulation within 7 days from diarrhea induction. Administration of PEA significantly improved diarrhea in a concentration-dependent manner (at 2 and 10 mg/kg, respectively); the antidiarrheal activity of PEA was significantly inhibited in the presence of PPARα antagonist (MK866), whereas PPARγ antagonist (GW9662) had no effect. The results are expressed as mean ± SEM of *n* = 5 experiments. ***p < 0.001 vs. vehicle group; °°*p <* 0.01 and °°°*p* < 0.001 vs. HIV-1 Tat group

Intracolonic administration of HIV-1 Tat-induced acute diarrhea, starting from day 1 and lasting up to 7 days post treatment; the severity of which was significantly improved by PEA, in a concentration-dependent fashion and in PPARα-dependent manner (Fig. 1). Interestingly, the antidiarrheal effect of PEA appeared to be specifically related to HIV-1 Tat administration since PEA treatment failed to significantly inhibit bisacodyl-induced diarrhea, even at the highest dose (Additional file 1).

**Fig. 2:**
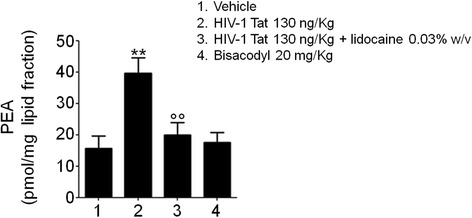
HIV-1 Tat administration increases endogenous PEA levels in submucosal plexi EGCs. As a consequence of HIV-1 Tat intracolonic administration, a marked increase of PEA produced in rat submucosal plexi was observed in comparison with vehicle group. Lidocaine (0.03% *w*/*v*) counteracted this HIV-1 Tat-induced effect, while bisacodyl treatment did not induce any significant changes in PEA levels. The results are expressed as mean ± SEM of *n* = 5 experiments. **p < 0.01 vs. vehicle group; °°*p* < 0.01 vs. HIV-1 Tat group

In order to provide a rational for the antidiarrheal effect of PEA, we measured its intracellular content in submucosal plexi after 7 days from the induction of the diarrhea. We found that HIV-1 Tat significantly increased the levels of endogenous PEA and that this increase was significantly inhibited by lidocaine (+ 147 and − 50% vs. vehicle group and HIV-1 Tat group, respectively; all *p <* 0.01; Fig. 2); in rats receiving bisacodyl, the tissue content of PEA was unaffected, likely suggesting that it is dependent by the activation of EGCs.

### HIV-1 Tat induces an inflammatory response through EGC activation that is inhibited by PEA

**Fig. 3:**
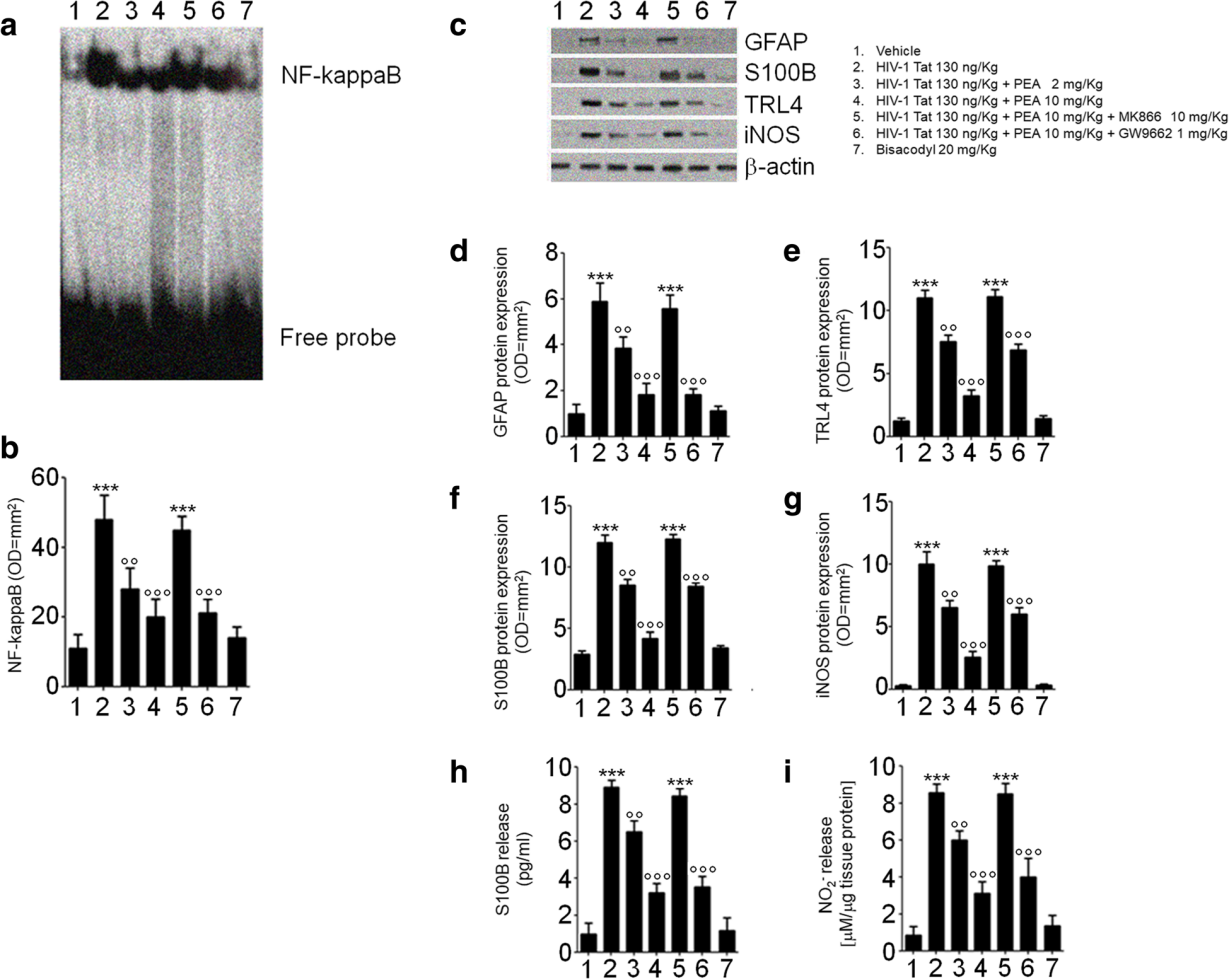
PEA inhibits HIV-1 Tat-induced inflammatory response in rat submucosal plexus-EGCs. HIV-1 Tat (130 ng/Kg) administration induced a significant increase of NF-*κ*B activation and GFAP, S100B, TLR, and iNOS protein expression, as well as S100B and NO_2_^−^ levels in submucosal plexi deriving from rats on day 7 after diarrhea induction. All HIV-1 Tat-mediated effects were inhibited by PEA (2–10 mg/kg) in a dose- and PPARα-dependent manner. Bisacodyl (20 mg/kg) did not show any significant effect. EMSA and immunoblot analysis showed, respectively, **a** NF-kappaB activation complex bands, **b** their densitometric quantification (OD = optical density in mm^2^), **c** representative immunoreactive bands of analyzed proteins, and **d**–**g** their respective densitometric quantification (normalized against the expression of the housekeeping protein β-actin; OD = optical densitometry in mm^2^). **h**, **i** Levels of S100B and NO_2_^−^ in submucosal plexi homogenates. The results are expressed as mean ± SEM of *n* = 5 experiments ***p < 0.001 vs. vehicle group; °°*p* < 0.01 and °°°*p* < 0.001 vs. HIV-1 Tat group

Administration of HIV-1 Tat induced a significant activation of NF-κΒ and resulted in an increased expression of GFAP, S100B, TLR4, and iNOS in rat submucosal plexi (Fig. 3, all *p <* 0.001 vs. vehicle group); significantly, the S100B and nitrite levels were also increased.

PEA treatment significantly reduced HIV-1 Tat-induced NF-κB activation, in a dose-dependent manner (− 42 and − 58%, *p <* 0.01 and *p <* 0.001); similarly, the activation of EGCs was inhibited by PEA, with the expression of GFAP (− 35 and − 69%), S100B (− 29 and − 65%), TLR4 (− 31 and − 70%), iNOS (− 35 and 74%), and the levels of S100B (− 27 and − 64%) and nitrite (− 30 and − 64%) being all significantly reduced (*p <* 0.01 and *p <* 0.001; at 2 and 10 mg/kg, respectively; Fig. 3). The ability of PEA to reduce HIV-1 Tat-induced EGC activation was significantly inhibited by MK866, but not by GW9662, suggesting that its effect was mediated by PPAR-α rather than by PPAR-γ. PEA failed to significantly affect the release of EGC-derived mediators in rats with bisacodyl-induced diarrhea (Fig. 3), and this finding suggests that its effect specifically targets HIV-1 Tat-induced diarrhea by acting on EGC-related activation.

**Fig. 4:**
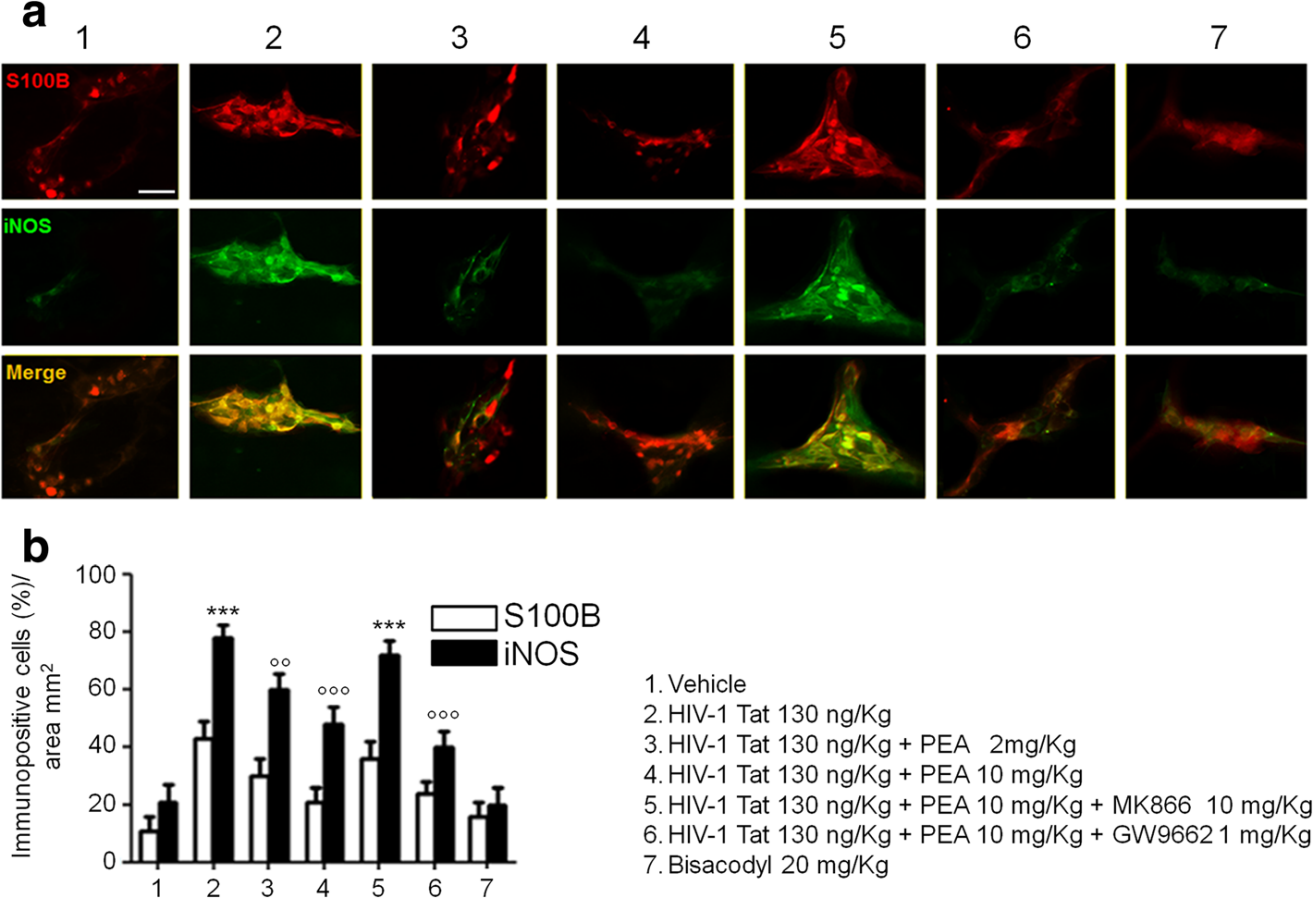
PEA reduces S100B/iNOS expression in submucosal plexi of HIV-1 Tat-treated rat. Immunofluorescence analysis showing that HIV-1 Tat (130 ng/Kg) induced a marked increase of S100B and iNOS expression in submucosal plexi that was reduced by PEA through a PPARα-dependent mechanism. Bisacodyl (20 mg/kg) administration failed to significantly affect S100B/iNOS co-expression. **a** The panel shows S100B (*red*) and iNOS (*green*) immunoreactivity and **b** their respective quantification (open and filled bars indicate S100B and iNOS expression, respectively). The results are expressed as mean ± SEM of *n* = 5 experiments. ***p < 0.001 vs. vehicle group; °°*p* < 0.01 and °°°*p* < 0.001 vs. HIV-1 Tat group. *Scale bar* = 20 μm

Further supporting the involvement of EGCs, a significantly higher S100B/iNOS expression was observed in the submucosal plexi preparation of HIV-1 Tat-treated rats, compared to vehicle group (+ 310 and + 260%, respectively, *p <* 0.001) (Fig. 4). Treatment with PEA (2–10 mg/kg) reduced the expression of both markers, in a concentration-dependent manner (− 28 and − 50%, − 26 and − 40% for S100B and iNOS, respectively; *p <* 0.01 and *p <* 0.001 vs. HIV-1 Tat; Fig. 4). Similar to what was reported above, the effect of PEA was inhibited by the presence of the PPARα-, but not PPARγ-antagonist, while bisacodyl administration failed to modify S100B/iNOS expression (Fig. 4).

### PEA treatment failed to improve HIV-1 Tat-induced diarrhea and EGC-associated neuroinflammation in PPARα^−/−^ mice

**Fig. 5:**
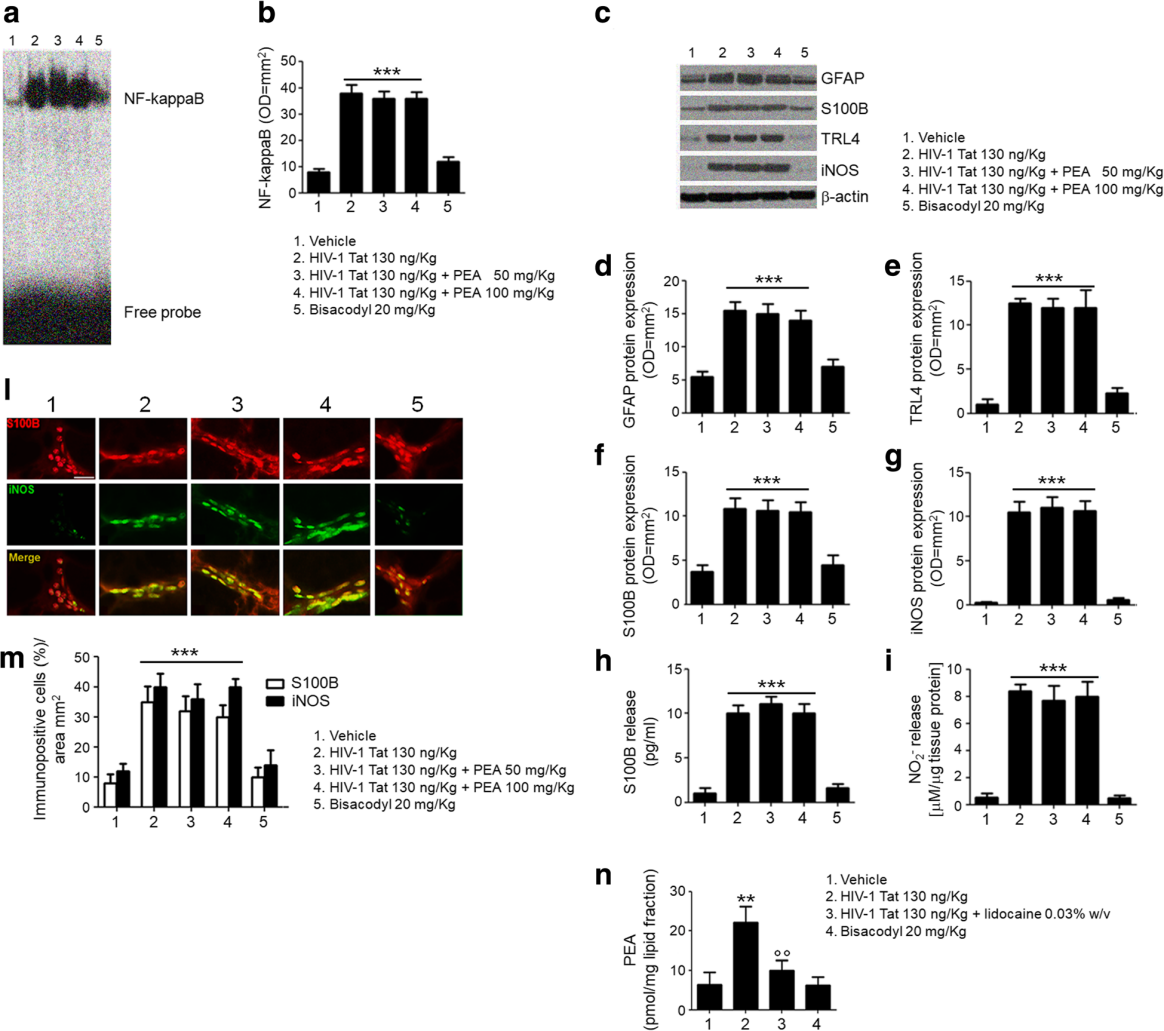
PEA fails to counteract submucosal plexus-EGC activation induced by HIV-1 Tat in PPARα^−/−^ mice. EMSA analysis showed marked upregulation of NF-kappaB in submucosal plexi at day 7 following HIV-1 Tat administration, compared to vehicle group. PEA was ineffective to reduce NF-kappaB activation in PPARα^−/−^ mice, while bisacodyl (20 mg/kg) did not induce NF-kappaB upregulation. **a** Representative NF-kappaB activation complex bands and **b** their densitometric quantification (OD = optical density in mm^2^). HIV-1 Tat significantly increased GFAP, S100B, TLR4, and iNOS protein expression and S100B and NO_2_^−^ levels in submucosal plexi isolated from PPARα^−/−^ mice in the same experimental conditions. Again, HIV-1 Tat-mediated effects were unaffected by PEA, while bisacodyl did not induce any change in the above investigated parameters. **c** Representative immunoreactive bands of analyzed proteins, **d**–**g** their respective densitometric quantification (normalized against the expression of the housekeeping protein β-actin; OD = optical densitometry in mm^2^) and **h**, **i** S100B and NO_2_^−^ levels. Immunofluorescence analysis showed an increased S100B/iNOS co-expression in submucosal plexus-EGCs at day 7 after HIV-1 Tat administration, vs. vehicle group. As expected, PEA was unable to counteract HIV-1 Tat activity and bisacodyl did not induce any S100B/iNOS upregulation. **l** S100B (red) and iNOS (green) immunoreactivity with **m** the respective quantification of S100B (open bars) and iNOS (filled bars) expression in the submucosal plexi. **n** HIV-1 Tat caused a marked increase of endogenous PEA content in submucosal plexi vs. vehicle group. Lidocaine inhibited HIV-1 Tat activity, whereas bisacodyl did not produce any alteration in endogenous PEA production. The results are expressed as mean ± SEM of *n* = 5 experiments. **p < 0.01 and ***p < 0.001 vs. vehicle group; °°*p* < 0.01 and °°°*p* < 0.001 vs. HIV-1 Tat group. *Scale bar* = 100 μm

Intracolonic administration of HIV-1 Tat protein induced acute diarrhea in PPARα^−/−^ mice, similar to what was observed in a subset of wild mice (data not shown) and in rats (Additional file 2); the diarrhea was associated with the increased activation of NF-kappaB of submucosal enteric glial cells and the upregulation of S100B/iNOS expression (Fig. 5). In line with the results obtained with the PPARα antagonist, treatment with PEA, even at highest doses, failed to significantly improve the diarrhea in PPARα^−/−^ mice and had no effect on the activation of EGCs induced by HIV-1 Tat (Fig. 5).

Bisacodyl treatment was able to induce the diarrhea in PPARα^−/−^ mice, but was unaffected by the administration of PEA (Fig. 5). Bisacodyl failed to modify the tissue content of endogenous PEA which was, conversely, significantly increased by HIV-1 Tat administration and inhibited by lidocaine (+ 250% vs. vehicle group, and − 55% vs. HIV-1 Tat group, respectively; all *p <* 0.01; Fig. 5).

## Discussion

Secretory diarrhea is a common clinical issue observed in nearly 60–80% of HIV-1 patients, and it is considerably widespread in third-world countries [28]. So far, HIV-1 Tat protein has been identified as the main responsible for the damage of intestinal mucosal, by promoting pro-oxidant and pro-apoptotic-mediated disruption of colonic epithelial cells and consequently the intestinal barrier integrity [5, 29]. More recently, it has been described that HIV-1 Tat protein has an additional effect on the nervous part of the gut, the ENS [7, 21, 30]. This direct action on the nervous system, which regulates many intestinal functions, causes abnormalities in neuronal excitability that, together with the release of pro-inflammatory cytokines in the intestinal milieu, contributes to gut dysfunction described in HIV patients [7].

Our results demonstrate that, beside its effect on enteric neurons, HIV-1 Tat protein also targets EGCs and mediates the overexpression of specific glial markers, as S100B and GFAP, in colonic submucosal plexus with a parallel increase in the expression of iNOS protein and pro-inflammatory signaling molecules (i.e., NO).

These final events occur via activation of the NF-kappaB-mediated cascade and TLR4 activation, two molecular pathways that are linked to each other during EGC activation [25].

The ability of the ENS to modulate virus-induced diarrhea was first reported by Lundgren et al., who showed that the inhibition of enteric nerves excitability was able to significantly inhibit the diarrhea induced by rotavirus [31]. Accordingly, we showed that lidocaine challenge was able to dampen symptoms and biochemical markers indicative for secretory diarrhea, further supporting the role of the ENS, as a whole, in mediating HIV-1 Tat-induced diarrhea. We here show that enteric glia cells take part in mediating the diarrhea induced by viral toxin and that their modulation is able to reduce HIV-1 Tat diarrheagenic effects by inhibiting the overexpression of S100B and iNOS and of the TLR4/NF-kappaB axis, respectively.

Furthermore, supporting the role of EGCs and their activation in HIV-1 Tat diarrhea, we also observed that when the secretory diarrhea was induced by a non-immunological stimulus (i.e., bisacodyl), no significant changes in glial network and markers were noticed.

Furthermore, we evaluated whether PEA was able to decrease glial activation and to improve the diarrhea, respectively. PEA has been recently showed to improve colonic inflammation through EGCs/TLR4-dependent PPARα activation [25]. Here, we demonstrated that PEA administration significantly and dose-dependently counteracted all diarrheal hallmarks in rodents, as shown by the decrease of stool frequency and weight, and by the rescue of water losses in colonic lumen.

According to our previous reports in human and animal models of intestinal inflammation, we also showed that the anti-diarrheal effect of PEA was selectively mediated by PPARα receptor activation [25, 32]. In fact, PPARα, but not PPARγ antagonist, significantly inhibited PEA effects, with this being further confirmed in a transgenic PPARα knockout model. Though HIV-1 Tat administration in these mice-induced secretory diarrhea, the treatment with PEA, even at high doses, did not evoke any anti-diarrheal effect, likely because the site of action was not expressed. However, it has to be noted that PPARα receptor sites are expressed by EGCs, as well as by enteric neurons, supporting that the anti-diarrheagenic effects of PEA are more likely to be the result of its synergistic effects on both neurons and glial cells.

According to our previous results, obtained in an experimental model of colitis [25], PEA was able to dampen EGC activation and the consequent overexpression of S100B and iNOS protein in the submucosal plexus isolated from colon. Moreover, we found that PEA, through the selective PPARα involvement, blocked the TLR4/NF-kappaB activation in the submucosal plexus of rats with HIV-1 Tat-induced diarrhea. These effects caused a decreased activation of EGCs with the reduction of S100B, GFAP, iNOS, and NO expression in the cell milieu. In PPARα^−/−^ mice, PEA failed to prevent HIV-1 Tat-induced EGC activation, further confirming that these effects are mediated by PPARα receptors.

Since its discovery, PEA has been believed to be an endogenous cannabinoid-like lipid able to suppress inflammatory responses in vitro [33]; moreover, PEA has been described to reduce gastrointestinal motility in mice model of colitis [34]. Also, during intestinal inflammation, PEA level increases, most probably as a protective response to mucosal damage [35]. The observation that PEA levels are higher in colonic mucosa of patients with ulcerative colitis and in experimental models of colitis strengths, the hypothesis that this endogenous compound may act as “on-demand modulator” of inflammatory processes in the gut [36]. Very interestingly, we found that in our models of HIV-1 Tat-induced diarrhea, the levels of endogenous PEA are significantly increased, while in the non-immunologic model of diarrhea obtained via bisacodyl administration, they remained unaltered. The fact that the endogenous level of PEA was significantly reduced by lidocaine indicates that, at least in our experimental conditions, PEA acts specifically by following the immune stimulus (HIV-1 Tat protein), behaving like a regulative ALIAmide and such action is intimately modulated by the ENS, and at least partly mediated by EGC activity.

## Conclusions

Although we cannot definitely rule out the role of enteric neuron dysfunction in mediating these effects, our results indicate that EGCs play a role in HIV-1 Tat-induced diarrhea and highlight the importance of these cells in regulating immune/inflammatory response featuring intestinal disturbance occurring in AIDS infection. We also demonstrated that PEA, by targeting HIV-1 Tat-induced neuroinflammatory responses, significantly modulates the diarrhea and that this occurs through the selective PPARα involvement. If confirmed by clinical trials in humans, our findings suggest that PEA, given its low cost and toxicological profile [37], might be regarded as promising tool that may integrate the current therapeutic approaches for treating a high-morbidity condition like diarrhea in HIV-infected patients.

## Additional files

Additional file 1: PEA failed to inhibit bisacodyl-induced diarrhea in rats. Bisacodyl (20 mg/kg) caused a significant increase of (**a**) daily defecation frequency, (**b**) average daily number of wet spots, and (**c**) fluid accumulation within 7 days from diarrhea induction, vs. vehicle group. PEA (2–10–50 mg/kg) resulted ineffective to exert any anti-diarrheal activity. The results are expressed as mean ± SEM of *n* = 5 experiments. ***p < 0.001 vs. vehicle group. (TIFF 1347 kb)

Additional file 2: PEA failed to improve diarrhea course in PPARα^−/−^ mice. HIV-1 Tat (130 ng/Kg)-induced diarrhea in PPARα^−/−^ mice, increasing (**a**) daily defecation frequency, (**b**) average daily number of wet spots, and (**c**) fluid accumulation within 7 days from diarrhea induction, vs. vehicle group. PEA (50–100 mg/kg) did not show any significant effect even at highest doses. The results are expressed as mean ± SEM of *n* = 5 experiments. ***p < 0.001 vs. vehicle group. (TIFF 580 kb)

## Abbreviations

AIDS: Acquired immunodeficiency syndrome
ALIAmides: Autacoid local inflammation antagonism amides
ANOVA: Analysis of variance
EDTA: Ethylenediaminetetraacetic acid
EGCs: Enteric glial cells
ELISA: Enzyme-linked immunosorbent assay
EMSA: Electrophoretic mobility shift assay
ENS: Enteric nervous system
GDNF: Glial cell-derived neutrophic factor
GFAP: Glial fibrillary acidic protein
GSNO: S-nitrosoglutathione
HIV-1: Human immunodeficiency virus-type 1
iNOS: Inducible nitric oxide synthase
LC-MS/MS: Liquid chromatography coupled to tandem mass spectrometry
NO: Nitric oxide
NO_2_^−^: Nitrite
PEA: Palmitoylethanolamide
PPARs: Peroxisome proliferator-activated receptors
PPARα: Peroxisome proliferator-activated receptor α
PPARα^−/−^: Peroxisome proliferator-activated receptor α knockout
PPARγ: Peroxisome proliferator-activated receptor γ
Tat: Trans-activating factor protein
TLR4: Toll-like receptor 4
TLRs: Toll-like receptors
