# Supplementary figures and images for: HIV-1 Tat-induced diarrhea is improved by the PPARalpha agonist, palmitoylethanolamide, by suppressing the activation of enteric glia
Source: J Neuroinflammation. 2018 Mar 24;15:94. doi: 10.1186/s12974-018-1126-4 (PMC5866515; doi:10.1186/s12974-018-1126-4)

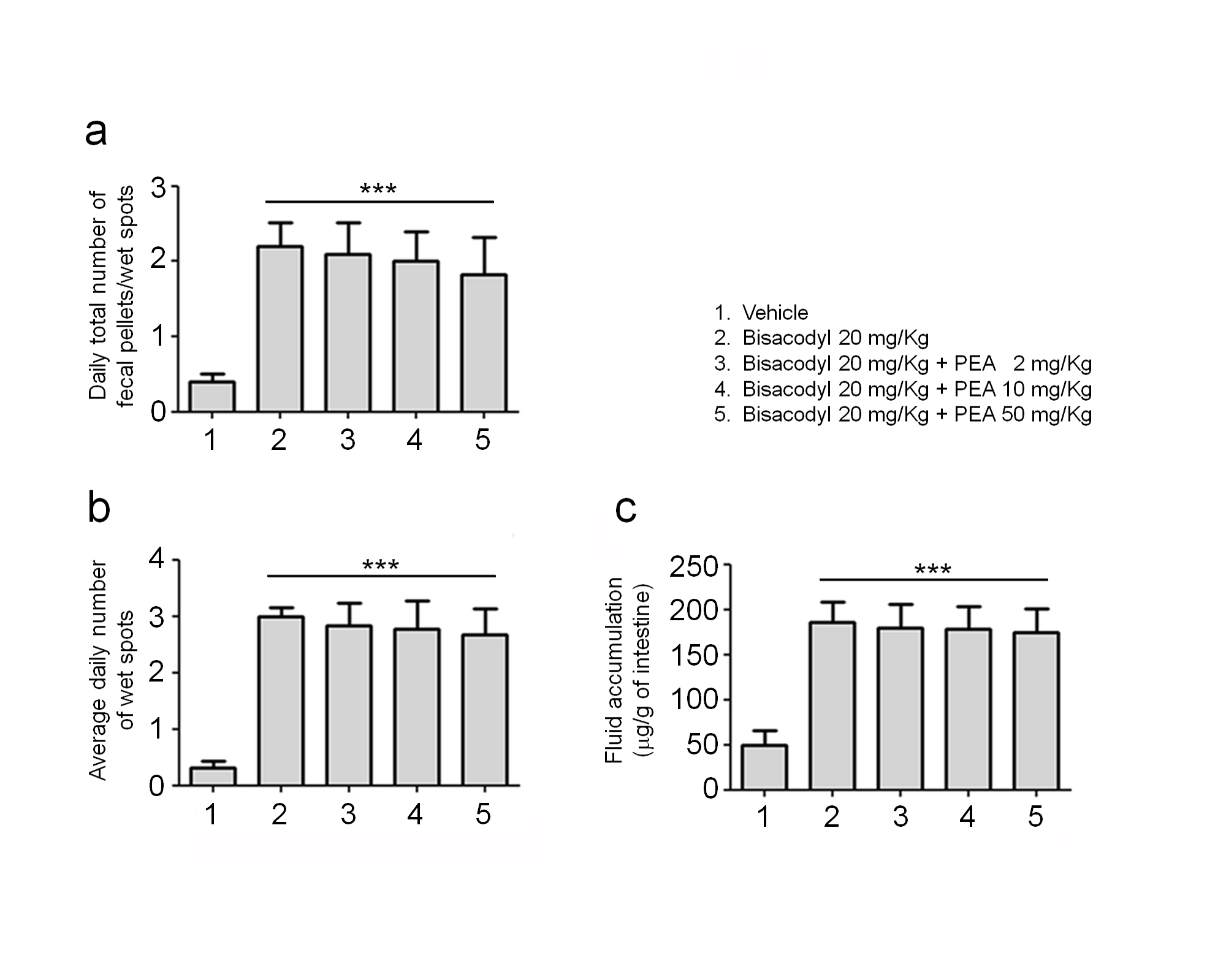

Supplement: Supplementary file 1 — PEA failed to inhibit bisacodyl-induced diarrhea in rats. Bisacodyl (20 mg/kg) caused a significant increase of (a) daily defecation frequency, (b) average daily number of wet spots, and (c) fluid accumulation within 7 days from diarrhea induction, vs. vehicle group. PEA (2–10–50 mg/kg) resulted ineffective to exert any anti-diarrheal activity. The results are expressed as mean ± SEM of n = 5 experiments. ***p < 0.001 vs. vehicle group. (TIFF 1347 kb) [file 12974_2018_1126_MOESM1_ESM.tif]

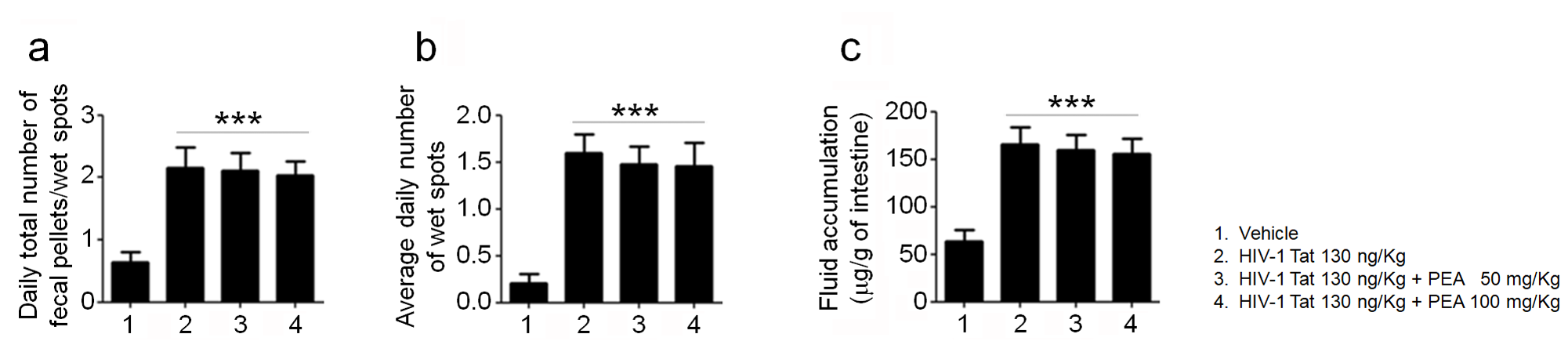

Supplement: Supplementary file 2 — PEA failed to improve diarrhea course in PPARα−/− mice. HIV-1 Tat (130 ng/Kg)-induced diarrhea in PPARα−/− mice, increasing (a) daily defecation frequency, (b) average daily number of wet spots, and (c) fluid accumulation within 7 days from diarrhea induction, vs. vehicle group. PEA (50–100 mg/kg) did not show any significant effect even at highest doses. The results are expressed as mean ± SEM of n = 5 experiments. ***p < 0.001 vs. vehicle group. (TIFF 580 kb) [file 12974_2018_1126_MOESM2_ESM.tif]
